# Biodegradable and Bioactive Carriers Based on Poly(betulin disuccinate-*co*-sebacic Acid) for Rifampicin Delivery

**DOI:** 10.3390/pharmaceutics14030579

**Published:** 2022-03-06

**Authors:** Daria Niewolik, Barbara Bednarczyk-Cwynar, Piotr Ruszkowski, Alicja Kazek-Kęsik, Grzegorz Dzido, Katarzyna Jaszcz

**Affiliations:** 1 Department of Physical Chemistry and Technology of Polymers, Silesian University of Technology, M. Strzody 9, 44-100 Gliwice, Poland; Katarzyna.Jaszcz@polsl.pl; 2 Department of Organic Chemistry, Poznan University of Medical Science, Grunwaldzka 6, 60-780 Poznan, Poland; bcwynar@ump.edu.pl; 3 Department of Pharmacology, Poznan University of Medical Science, Rokietnicka 5a, 60-806 Poznan, Poland; pruszkowski@gmail.com; 4 Department of Inorganic Chemistry, Analytical Chemistry and Electrochemistry, Silesian University of Technology, B. Krzywoustego 6, 44-100 Gliwice, Poland; alicja.kazek-kesik@polsl.pl; 5 Biotechnology Centre, Silesian University of Technology, B. Krzywoustego 8, 44-100 Gliwice, Poland; 6 Department of Chemical Engineering and Process Design, Silesian University of Technology, M. Strzody 7, 44-100 Gliwice, Poland; grzegorz.dzido@polsl.pl

**Keywords:** betulin, polyanhydrides, biodegradable polymers, rifampicin, drug delivery systems

## Abstract

This paper describes the preparation and characterization of polymer-drug systems based on polymeric microspheres obtained from poly(betulin disuccinate-*co*-sebacic acid). The active compound that was coupled to the betulin-based carriers was rifampicin (RIF), an ansamycin drug used in the treatment of tuberculosis. Poly(betulin disuccinate-*co*-sebacic acid) microspheres were prepared using a solvent evaporation technique from copolymers obtained by polycondensation of betulin disuccinate (*DBB*) and sebacic acid (*SEB*). The content of sebacic acid in the copolymers was 20, 40, 60 and 80 wt%, respectively. Small and large rifampicin-loaded microspheres were obtained for each of the copolymers. The initial amount of drug was 10, 30 or 50 wt%, based on the weight of the polymer. Particles obtained in this study were round in shape with diameter in the range of 2–21 μm and of orange to red colour originating from rifampicin. The RIF encapsulation efficacy varied from 7% to 33%. Drug loading varied from 2% to 13% and increased at a higher RIF ratio. The highest degree of drug loading was observed for large particles, in which the initial amount of drug (at the particle preparation stage) was 50 wt%. Microspheres prepared from betulin-based polyanhydrides may have significant applications in drug delivery systems. The concentration of loaded drug was enough to obtain bactericidal effects against reference S. Aureus ATCC 25923 bacteria.

## 1. Introduction

Betulin, a pentacyclic triterpene, is a natural compound found in the outer layer of birch bark. Due to its bifunctionality, it can be used to obtain polymers. In recent years, polymers derived from natural monomers (e.g., lactic acid, furans or terpenes) have become more desirable because they can replace petroleum-based raw materials [1]. Polymers obtained from renewable raw materials are in demand in many industries, including pharmaceuticals. Betulin, just like other natural triterpenes, exhibits a broad spectrum of biological activity [2,3]. The biological activity of betulin is well documented, and has been proven to be also effective in both oncological [4] and bacterial [5] lung diseases. Betulin exhibits synergistic effects with other biologically active compounds, such as acyclovir (against herpes simplex viruses) [6], gamma-cyclodextrin derivatives (reduction of the cell proliferation and induced differentiation and cell death in melanoma B164A5 cells) [7] and 5-fluorouracil (treatment of ovarian cancer) [8] as well as synergistic therapeutic effects in lung cancer treatment [5,9,10].

Betulin disuccinate (*DBB*), similarly to betulin, also exhibits a broad spectrum of biological activity, including anticancer, while being non-toxic to normal cells [3,11,12,13]. Due to the presence of two carboxyl groups, *DBB* is an ideal substrate for the preparation of polyanhydrides. Biodegradable polyanhydrides are useful materials for controlled drug delivery systems. They have hydrophobic backbones with hydrolytically unstable anhydrides, which may hydrolyze in aqueous medium to dicarboxylic acids and are completely eliminated from the body within a short period of time [14,15]. These hydrophobic polymers can be used as controlled release carriers for short-lived drugs due to their surface erosion causing sustained drug release over an extended period of time [16].

Polyanhydrides have been investigated as potential vehicles for biologically active compounds, such as chemotherapeutics [11,17,18], antibiotics [19,20], or anaesthesis [21,22]. Few drug delivery systems based on polyanhydrides have reached the clinical stages. The most well-known polyanhydride device used in medicine is Gliadel, based on 20:80 poly[(1,3,bis-*p*-carboxyphenoxypropane)-*co*-(sebacic anhydride)] (CPP-SA). CPP-SA has been approved by the FDA for use in medicine in delivering carmustine for the treatment of brain cancer [23]. In our previous publications, we described the synthesis and characterization of polyanhydrides based on betulin disuccinate and other diacid comonomers that exhibited anticancer activity [24,25,26]. These polymers release *DBB* as a result of hydrolytic degradation in their physiological condition; thus, they can be used as a polymeric prodrug. Due to their biodegradability and non-toxicity, they are also ideal candidates for carriers of other biologically active substances. Until now, no reports have appeared regarding preparation of polymer-drug systems based on polyanhydrides from betulin derivatives and thus there are no reports about their use in controlled drug delivery systems.

The aim of this work was the preparation and characterization of polymer-drug systems based on polymeric microspheres obtained from polyanhydrides composed of betulin disuccinate and sebacic acid. The active compound that was coupled to the betulin-based carriers was rifampicin, an ansamycin drug used in the treatment of tuberculosis. Rifampicin (RIF) is one of the most powerful antibiotics mainly used for the treatment of tuberculosis, as one of the first-line drugs recommended by the World Health Organization [27]. However, RIF has many disadvantages including: short biological half-life, poor water solubility and bioavailability and many side effects. RIF bactericidal activity is proportional to its concentration at the target size; thus, low dissolution of RIF in biological liquids can limit its ability to reach the required concentration [27,28]. Encapsulation of RIF in a carrier (e.g., in microspheres) limits adverse side effects and enhances the therapeutic activity of RIF [28].

The objective of the present study was to evaluate the potential of biodegradable poly (betulin disuccinate-*co*-sebacic acid) as a rifampicin carrier. Depending on the composition of polyanhydride, the difference in degradation behaviour can be used to influence release profiles of rifampicin. Taking into account betulin’s low toxicity, as well as its protective effects from lung injury [5,29], botulin-based polymers look promising as carriers of RIF for the treatment of lung diseases.

## 2. Materials and Methods

### 2.1. Materials

Betulin disuccinate (obtained in the laboratory according to the procedure described in the article [26]), acetic anhydride (POCh S.A., Gliwice, Poland), sebacic acid (ACROS Organics, Geel, Belgium), rifampicin (Biosynth Carbosynth, Berkshire, UK), poly(vinyl alcohol) (*M_w_* = 88,000 g/mol, 88% hydrolyzed) (ACROS Organics, Geel, Belgium), and methylene chloride (Chempur, Piekary Śl., Poland) were used as supplied.

### 2.2. Prepolymer and Polymer Synthesis

Polyanhydrides were obtained by polycondensation of betulin disuccinate and sebacic acid (SA) according to the procedure described earlier [24,25,26]. Betulin disuccinate and sebacic acid were mixed in defined ratios (Table 1) and refluxed in acetic anhydride (1:10, *w*/*v*) under nitrogen flow for 40 min, forming prepolymers.

The obtained prepolymers were then heated at 150 °C for 2 h under a vacuum (0.1 mm Hg) in order to obtain copolymers (polyDBB_SEB) with a yield of over 90%.

### 2.3. Formulation of Microspheres

The obtained polyanhydrides were formulated into blank- and drug-loaded microspheres using the emulsion solvent evaporation method, according to previous reports [24,25].

#### 2.3.1. Blank Microspheres

The polyDBB_SEB solution in methylene chloride (50 mg/mL) was emulsified in aqueous solution (1% *w*/*w*) of poly(vinyl alcohol) using ULTRA-TURRAX T18 homogenizer for 30 s. The speed of the homogenizer was 3000 rpm for large particles and 18,000 rpm for small particles. The emulsion was stirred with a magnetic stirrer at 1100 rpm at room temperature for 3 h to evaporate the organic solvent. After that, microspheres were collected by centrifugation at 5000 rpm for 5 min., washed three times with distilled water, lyophilized and stored in a freezer.

#### 2.3.2. Rifampicin (RIF) Loaded Microspheres

The procedure for preparation of rifampicin loaded microspheres was similar to that used for the preparation of blank particles. Rifampicin (10, 30 or 50% *w*/*w* in respect to the mass of polymer) was dissolved in methylene chloride polymer solution and then the organic phase was emulsified in aqueous solution (1% *w*/*w*) of PVAl solution. The solidification and isolation of drug loaded microspheres were performed similarly to blank microspheres. The obtained RIF-loaded microspheres are listed in Table 2.

### 2.4. Characterization of Polyanhydrides and Microspheres

#### 2.4.1. Nuclear Magnetic Resonance (NMR) Spectroscopy

^1^H NMR and ^13^C NMR spectra of polymers were recorded on a Varian 600 MHz spectrometer using CDCl_3_ as the solvent and TMS as an internal standard. 

Molecular weights were calculated from the ^1^H NMR spectra, based on Equations (1)–(6).

(1) $$M_{w}=n_{DBB}M_{DBB}+n_{SEB}M_{SEB}+M_{T}$$

(2) $$I_{\left[1H\right]DBB}=\left(I_{C29-Ha}+I_{C29-Hb}+I_{C28-Ha}+I_{C28-Hb}+I_{C3-H}+I_{SAc}+I_{E}\right)/13$$

(3) $$I_{\left[1H\right]T}=I_{T}/6$$

(4) $$I_{\left[1H\right]SEB}=\left(I_{\delta =2.52-2.40\;\mathrm{ppm}}-I_{\left[1H\right]DBB}\right)/4$$

(5) $$n_{DBB}=\frac{I_{\left[1H\right]DBB}}{I_{\left[1H\right]T}}$$

(6) $$n_{SEB}=\frac{I_{\left[1H\right]SEB}}{I_{\left[1H\right]T}}$$

where: *M_DBB_*_—_molar mass of *DBB* unit (642.86 g/mol), *M_*SEB*_*—molar mass of *SEB* unit (202.25 g/mol), *M_T_*—molar mass of end groups (102 g/mol) *I_*[1H]*DBB_*—intensity of one *DBB* proton, *I_*[1H]*SEB_*_—_intensity of one *SEB* proton, *I_*[1H]*T_*—intensity of one proton of end groups, *I_δ_* *= _2.52–2.40 ppm_*_—_intensity of signal of five protons (C_28_-H_a_ and -CH_2_C(O)OC(O)- in *SEB*), *I_T_*_—_intensity of signal of terminal groups (δ = 2.24 ppm), *I_(C29-Ha)_* and *I_(C29-Hb)_*—intensity of signal assigned to methylene protons at the double-bonded carbon (δ = 4.68 and 4.59 ppm), *I_(C3-H)_*—intensity of signal assigned to metine proton in the ring of betulin (δ = 4.50 ppm), *I_SAc_*—intensity of signal assigned to methylene protons in the anhydride moiety (δ = 2.82–2.78 ppm) and *I_E_*—intensity of signal assigned to methylene protons in the ester moiety (δ = 2.71–2.64 ppm).

#### 2.4.2. Gel Permeation Chromatography (GPC)

Molecular weights (*M_n_*) and molecular weight distributions (DP) of polyanhydrides were determined by gel-permeation chromatography (GPC) using Agilent Technologies Infinity 1260 chromatograph equipped with a refractive index detector and calibrated with linear polystyrene standards (580–300,000 g/mol). The measurements were carried out in methylene chloride as the solvent with a flow rate of 0.8 mL/min.

#### 2.4.3. Fourier Transform Infrared Spectroscopy (FT-IR)

In order to check for any chemical interaction between rifampicin and copolymers, FT-IR analysis was carried out. The tested samples included selected rifampicin-loaded microspheres, rifampicin, selected blank microspheres and their physical mixture. FT-IR spectra were recorded using a PerkinElmer Spectrum Two Spectrometer. Spectra were recorded at 128 scans per spectrum in the range of 4000–400 cm^−1^ with a resolution of 1 cm^−1^.

#### 2.4.4. Differential Scanning Calorimetry (DSC)

Thermal analyses of rifampicin, polyanhydrides, rifampicin-loaded microspheres and blank microspheres were carried out using a 822 ^e^ DSC Mettler Toledo differential scanning calorimeter. Samples of about 3 mg were tested in a temperature range from −60 °C to 250 °C at a heating rate of 10 °C/min.

#### 2.4.5. SEM Analysis

The morphological characterization of microspheres was carried out using a Phenom ProX scanning electron microscope (SEM) at an accelerating voltage of 10 kV. Samples were coated with a 10 nm gold layer under vacuum using sputter coater Quorum Q150R ES.

#### 2.4.6. Particle Size and Particle Size Distribution

An optical microscope DELTA Optical ME 100 was used to determine mean diameters of obtained microspheres. The diameters were measured on the microscope images using PHAMIAS 2003 v.1.3 B software and then, number and volume mean diameters (*D_n_* and *D_v_*), (Equations (7) and (8)), standard deviation (S) and dispersity index (*D_v_*/*D_n_*) were calculated.

(7) $$D_{n}=\frac{\sum^{}N_{i}D_{i}}{\sum^{}N_{i}}$$

(8) $$D_{v}=\frac{\sum^{}N_{i}D_{i}^{4}}{\sum^{}N_{i}D_{i}^{3}}$$

where *Ni* is the number of particles having diameter *Di*.

#### 2.4.7. Zeta Potential Measurements

Zeta potential (ZP) measurements for small blank and rifampicin-loaded microspheres (with the greatest amount of RIF) were carried out using the Zetasizer Z90 (Malvern, UK). Before measurements, particles were dispersed in distilled water. Measurements were conducted at a pre-set temperature of 25 °C, reached after thermostating. Zeta potential was determined five times for each sample, with the final value being the arithmetic mean of the readings.

### 2.5. Hydrolytic Degradation of Copolymers

Hydrolytic degradation experiments for disc shaped samples (10 mm diameter, 2 mm thickness and 0.1 g weight) were performed in a phosphate buffer solution of pH 7.4 (PBS) at 37 °C, according to the procedure described earlier [26]. The hydrolytic degradation was monitored by recording the content of anhydride groups in test samples (for *DBB* and *SEB* segments) and the *DBB* to *SEB* ratio.

The ratio of anhydride groups to the sum of anhydride and ester groups for *DBB* or *SEB* segments was calculated using the Equations (9) and (10).

(9) $${\left(\frac{A}{A+E}\right)}_{DBB}=\frac{I_{SAc}}{I_{SAc}+I_{E1}}$$

where: *I_Sac_*—intensity of the signal of methylene protons in the anhydride moiety (δ = 2.82–2.78 ppm) and *I_E1_*—intensity of the signals of methylene protons in the ester moiety (δ = 2.71–2.64 ppm).

(10) $${\left(\frac{A}{A+E}\right)}_{SEB}=\frac{I_{A}-I_{\left[1H\right]DBB}}{(I_{A}-I_{\left[1H\right]DBB})+I_{E2}}$$

where *I_A_*—intensity of the signal of methylene protons in the anhydride moiety (δ = 2.52–2.40 ppm), *I_*[1H]*DBB_*—intensity of the signal of one *DBB* proton and *I_E2_*—intensity of the signals of methylene protons in the ester moiety (δ = 2.40–2.30 ppm).

The ratio of the *DBB* segment to the *SEB* segment in the polyanhydride (*DBB*/*SEB*) was calculated using the Formula (11).

(11) $$\left(\frac{DBB}{SEB}\right)=\frac{I_{\left[1H\right]DBB}}{I_{\left[1H\right]SEB}}$$

where *I_*[1H]*DBB_*—intensity of one *DBB* proton and *I_*[1H]*SEB_*—intensity of one *SEB* proton (calculated according to Equations (2) and (4)).

### 2.6. In Vitro Rifampicin Release

Drug release studies were performed in PBS (release medium) at 37 °C under static conditions. Accurately weighted amounts of microspheres (around 5 mg) containing rifampicin were placed in vials and suspended in 1.5 mL of PBS. The vials were incubated at 37 °C. After a defined period of time (1 h to 30 days), the samples were centrifuged and 1 mL of supernatant was removed. To maintain a constant volume of the release medium, 1 mL of fresh PBS was added. Vials were then briefly vortexed to resuspend the microspheres. The concentration of rifampicin in the supernatant was determined with UV/Vis analysis at λ = 470 nm, according to the standard curve of RIF in PBS. Dissolution curves were determined from triplicate runs.

Cumulative release (*Su*) of rifampicin was calculated in respect to mass of microspheres, according to Equations (12) and (13).

(12) $$Su=\sum^{}m_{SMn-1}+1.5\;m_{SMn}$$

(13) $$m_{SMn}=\left(C\times r\right)/m_{m}$$

where: *m_SMn_*—the mass of rifampicin in the n-th sample of supernatant (taken after a specified time) with respect to 1 mg of microspheres [μg/mg]; *C*—concentration of rifampicin in a buffer solution in the *n*-th sample [μg/mL]; *r*—dilution of the buffer solution used for the analysis of UV; *m_m_*—weight of microspheres [mg].

Absorbance spectra of rifampicin obtained from fresh solution and supernatant sampled at various times during the release experiment were identical in shape, indicating that there was no degradation of model compounds during the release period.

### 2.7. Estimation of Drug Loading and Encapsulation Efficiency

Total amount of RIF contained in microspheres was directly determined by dissolving 5 mg of loaded microspheres in chloroform and subsequent determination of amount of rifampicin in organic solutions by UV at λ = 334 nm.

The actual loading of model compound (*L_A_*) encapsulation efficiency (*EE*) and drug loading (*DL*) were calculated from the weight of the initial drug loaded microspheres and the amount of model compounds used and incorporated, according to Equations (14)–(16). Samples were run in triplicate.

(14) $$m_{SMn}=\left(C\times r\right)/m_{m}$$

(15) $$\%\;EE=\left(\frac{L_{A}}{L_{Th}}\right)\times 100$$

(16) $$\%\;DL=\left(\frac{L_{A}}{1000}\right)\times 100$$

where: *L_A_*—actual loading of rifampicin [μg/mg]; *m_SMn_*—the weight of rifampicin [μg] encapsulated in microspheres; *m_m_*—the weight of rifampicin-loaded microspheres [mg]; *L_Th_*—theoretical loading of rifampicin (*L_Th_* = 10, 30 or 50 μg/mg).

### 2.8. Drug Release Kinetics

The kinetics and mechanism of rifampicin release from poly(betulin disuccinate-*co*-sebacic acid) microspheres were evaluated by fitting the in vitro drug release data to four kinetics models: zero order, first order, Higuchi, and Hixson-Crowell and Korsmeyer-Peppas models. Zero order kinetics (Equation (17)) shows the linear relationship between amount released and time. The ideal method of drug release is to achieve a prolonged pharmacological action. First order kinetics (Equation (18)) describes the release from systems, where the release rate is proportional to the amount of drug remaining to be released. The Higuchi model (Equation (19)) describes drug release from an insoluble matrix as a diffusion process based on Fick’s law, square root time-dependent. The Korsmeyer-Peppas model (Equation (20)) is used to analyze drug release from polymeric systems when the release mechanism is not well known or when more than one type of release phenomenon is involved [28,30,31,32].

(17) $$M_{t}=M_{0}+K_{0}t$$

(18) $$\ln M_{t}=\ln M_{0}+K_{1}t$$

(19) $$\ln M_{t}=\ln M_{0}+K_{1}t$$

(20) $$\frac{M_{t}}{M_{\alpha }}=K_{k}t^{n}$$

where: *M_t_*—cumulative amount of drug released in time t; *M_0_*—initial amount of drug; *M_t_*/*M_α_*_—_fraction of the drug release at time *t* and *K_0_, K_1_, K_H_* and *K_k_* are release rate constant for mentioned kinetic models.

Regression analysis was performed to obtain R^2^ (coefficient of correlation) values of the linear curves, rate constants and n-values (diffusion exponent obtained from the slope of the Korsmeyer-Peppas plots).

### 2.9. Antibacterial Properties

The microbial tests of selected samples (blank and the respective rifampicin-loaded samples) were carried out using reference *Staphylococcus aureus* (ATCC 25923) bacterial strains. The antibacterial properties of samples were evaluated using the solution obtained from microspheres after 24 h of immersion in PBS (5 mg of sample in 6 mL of PBS) at 37 °C. Before testing, the samples were centrifuged (18,000 rpm, 3 min, 5 °C) and the extracts were filtered through a sterile 0.2 μm nylon filter. The bacterial strains were precultured in the TSB (Triptic Soy Broth) culture medium at 37 °C for 18 h (incubator POL-EKO, Wodzisław Śl., Poland). Then, 1 mL of TSB of the filtered sample (solution obtained from microspheres) was added to 1 mL of TSB with 5 × 10^8^ CFU/mL of the bacteria (*Staphylococcus aureus* ATCC 25923), and the optical density (OD) was measured using a densitometer (Densilameter, Erba Lachema, Brno, Czech Republic). The samples were cultured at 37 °C for 18 h, and the OD was measured again. The results were presented as an average value obtained according to the McFarland scale. The experiment was carried out using three independent samples.

For samples where no increase in O.D. value was determined, 100 μL of the previously filtered solution was placed onto agar plates (Muller-Hinton agar, Diag-Med Poland). The samples were cultured at 37 °C for 18 h (incubator POL-EKO, Poland).

In the next stage, the inhibition zones of bacteria were determined. Before testing, the suspension of *Staphylococcus aureus* ATCC 25923 in TBS (~5 × 10^8^ CFU/mL) was prepared. The bacteria were spread onto agar plates (Muller-Hinton agar, Diag-Med Poland). Then, a 5-mm hole was cut out from the middle of the agar plate. Next, 100 μL of sample solution was placed into this hole. Agar plates were incubated at 37 °C for 18 h, after which the inhibition zones were measured.

## 3. Results

### 3.1. Betulin-Based Polyanhydrides Synthesis and Characterization

**Figure 1:**
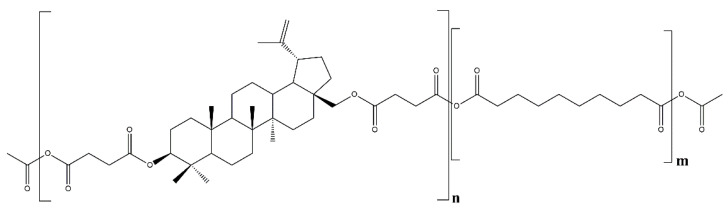
Structure of polyDBB_SEB.

A series of polyanhydrides were obtained by melt polycondensation of betulin disuccinate (*DBB*) and sebacic acid (SA) with the use of acetic anhydride. Previously, we have described the synthesis and characterization of a betulin disuccinate homopolymer (polyDBB) [26] and its copolymers with dicarboxylic derivatives of poly(ethylene glycol) (polyDBB_PEG) [27]. Obtained polyanhydrides exhibited anticancer activity against a various cancer cell lines. In this study, sebacic acid was selected as a comonomer to increase the crystallinity of polymers. The content of SA in copolymers ranged from 20 to 80 wt%. The chemical structure of polyDBB_SEB shown in the Figure 1, was confirmed by spectroscopic methods FT-IR, ^1^H NMR and ^13^C NMR. The presence of two characteristic bands at 1724 cm^−1^ and 1827 cm^−1^ in the carbonyl region of the FT-IR spectra affirmed that polyanhydrides were obtained.

**Figure 2:**
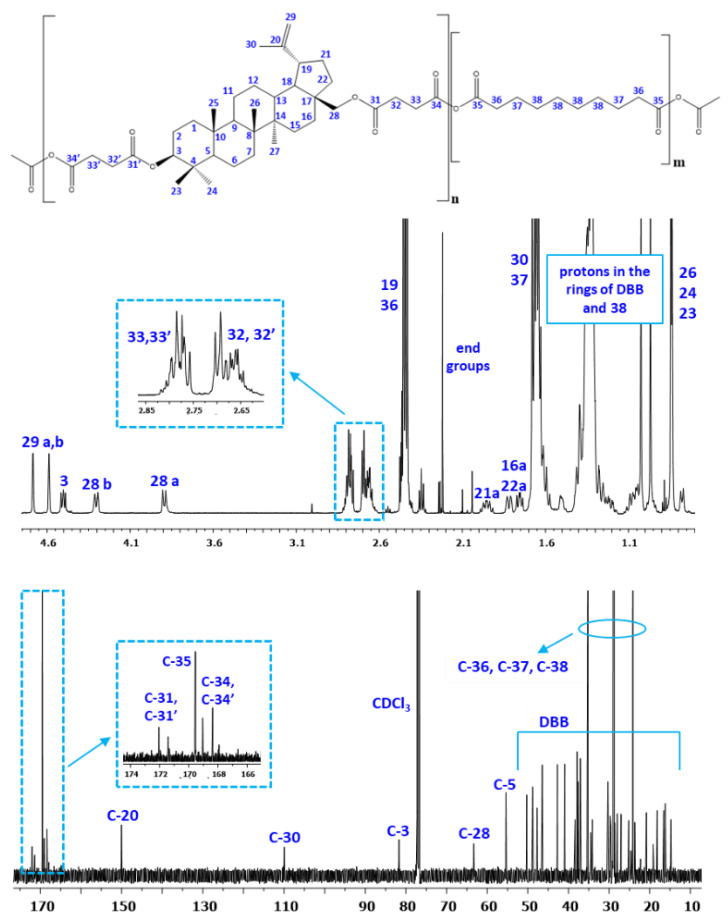
^1^H NMR and ^13^C NMR spectra of polyDBB_SEB.

Figure 2 shows the typical ^1^H NMR and ^13^C NMR spectra of the copolymers. In previous work [26] we presented detailed description of all signals appearing in IR and NMR spectra.

The presence of the signals at δ = 2.82–2.78 ppm (33 and 33′ in the Figure 2) and at δ = 2.48–2.40 ppm (32 and 32′ in the Figure 2) in the ^1^H NMR spectra, and at δ = 169.55 and 167.94 ppm (35, 34 and 34′ in Figure 2) in the ^13^C NMR spectra confirmed the formation of the polyanhydrides. The presence of the signals at δ = 2.52–2.40 ppm (C_37_-H_2_) and 1.45–1.35 ppm (C_38_-H_2_) in ^1^H NMR and δ = 35.22 ppm (C), δ = 28.95 ppm (C), δ = 28.75 ppm (C) and δ 24.14 ppm (C) in ^13^C NMR spectra confirmed the presence of the SA in polyanhydrides.

The rest of the ^1^H NMR and ^13^C NMR signals assigned to the relevant protons and carbons of the repeating unit of the *DBB* segments were discussed in detail in our previous work [24].

The molecular weight of the copolymers was calculated from ^1^H NMR and determined by GPC. The molecular weights values are summarized in Table 3.

**Figure 3:**
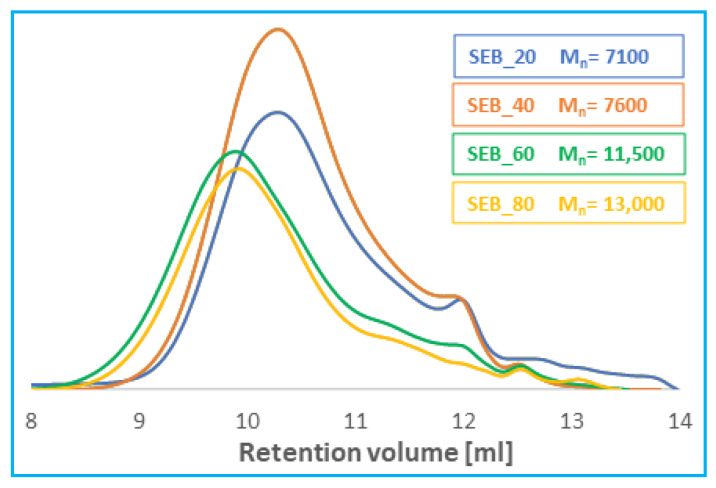
Gel-permeation chromatography (GPC) chromatograms of *DBB*-*SEB* polyanhydrides.

The molecular weight of polyanhydrides calculated from ^1^H NMR ranged from 11,000 to 15,000 and were higher than what was determined by GPC (Mn = 7100–13,000) (Figure 3). The molecular weight determined by GPC showed relatively broad dispersity. DP were in the range of 2.24–4.41. Molecular weights of copolymers increases with the increase of SA content in polymer. The highest average Mn was observed for polymer containing 80 wt%.

The thermal properties of the copolymers, such as glass transition temperature (Tg), melting temperature (Tm) and heat of fusion of melting process (ΔHm), were investigated using the DSC method (Table 3).

Copolymers containing more *DBB* were completely amorphous. No crystallinity was observed within the temperature range of –60 to 250 °C. Even the low SA content in the copolymers reduces the Tg, as compared to polyDBB (Tg = 124 °C), which affects the physical characteristics of the copolymers. Increasing the SA content in polymers above 40 wt% increases the crystallinity of obtained polyanhydrides. DBB_SEB_60 and DBB_SEB_80 were crystalline, with two melting peaks at 41.1 and 68.0 °C for DBB_SEB_60 and a sharp melting peak at 80.3 °C for DBB_SEB_80. The heat of fusion of polyanhydrides containing 60–80 wt% of SA increased with the increase of SA content (from −9.03; −27.27 for DBB_SEB_60 to −74.96 for DBB_SEB_80), which indicated higher crystallinity of DBB_SEB_80 compared to DBB_SEB_60.

Table 4 summarizes the solubility results for the polyDBB_SEB samples. Copolymers were found to be insoluble in water, ethanol, diethyl ether and hexane, but could be dissolved in toluene, methylene chloride, chloroform and THF. They were also partially soluble in acetone and DMSO.

In previous work [26], we presented the results concerning the cytostatic activity of polyDBB_SEB. Polyanhydrides were studied to determine their cytostatic activity against selected cancer cell lines. Cell lines representing cervix, breast, lung, liver, central nervous system and nasopharynx tumors were used in these studies to find the concentrations causing inhibition of cell growth in the culture by 50% (IC_50_). Human dermal fibroblast (HDF) cell lines were used as non-proliferative cells to compare results from human cancer cell lines and to establish selectivity between cancer and non-cancer cells. Cytostatic tests indicated the effectiveness of obtained copolymers in the inhibition of growth of cancer cells (IC_50_ < 12 μg mL^−1^), with limited cytotoxicity towards normal cells. The results confirmed that the cytostatic activity is dependent on the amount of *DBB* and increase with the increase of *DBB* content in polyanhydrides. Polyanhydrides containing 80 wt% of *DBB* (DBB_SEB_20) showed the highest cytostatic activity (IC_50_ values in range of 4.15 to 4.99 μg mL^−1^, depending on the type of cancer cell line). The selectivity index, defined as the ratio of IC_50_ values between the normal and cancer cell lines (IC_50HDF_/IC_50cancer cell lines_), was the highest for DBB_SEB_20, with values to 1.76. Copolymers based on betulin disuccinate and *SEB* can be used as degradation-based delivery systems for *DBB* or combined with other chemotherapeutic agents can lead to a synergistic therapeutic effect in cancer treatment. Due to the low toxicity of polyDBB_SEB towards normal cells and the confirmed protective effects of betulin on lung injury [8,30], such polymers can also be used as drug delivery carriers for the treatment of lung diseases.

### 3.2. Hydrolytic Degradation of Polymers (In Vitro Degradation and Stability)

**Figure 4:**
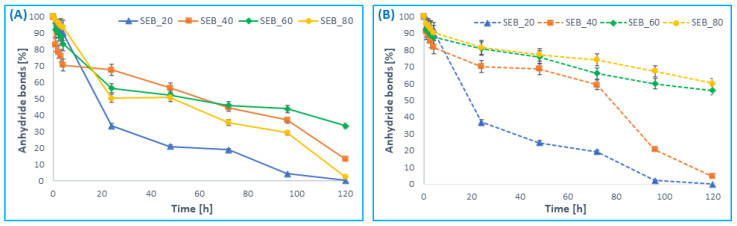
Anhydride bond loss of *DBB*-*SEB* polyanhydrides, for *DBB* segments (**A**) and SA segments (**B**) during hydrolytic degradation in phosphate buffer conducted at 37 °C (n = 3, error bars, standard deviation).

Hydrolytic degradation was carried out in PBS (pH 7.4) at 37 °C. As a result, the disappearance of anhydride bonds in polyanhydrides was observed (Figure 4). ^1^H NMR spectra of lyophilized post-degradation buffer solution indicated the presence of betulin disuccinate and sebacic acid among the degradation products.

The insertion of *SEB* comonomer to polyanhydrides accelerated the hydrolytic degradation process of polymers, compared to the homopolymer obtained from *DBB*. The complete degradation of polyDBB was observed in about 14 days [24], whereas DBB_SEB_20 degraded completely within 5 days (Figure 4). The degradation rate of copolymers grows with the increase in content of betulin disuccinate in polyanhydrides. The degradation process of *SEB* segments (Figure 4B) in polyanhydrides containing 20 and 40 wt% of SA was faster compared to polyanhydrides containing 60 and 80 wt% of SA. This is caused by the difference in crystallinity and in the chemical structure of copolymers. DBB_SEB_20 and DBB_SEB_40, which are amorphous, degraded almost completely within five days. The crystalline copolymers—DBB_SEB_60 and DBB_SEB_80—degrade relatively slowly (after five days of degradation, the disappearance of anhydride bonds in samples was about 35–40%). However, the degradation process of *DBB* segments (Figure 4A) in DBB_SEB_20 and DBB_SEB_80 was faster compared to copolymers containing 40 and 60 wt%. It can be concluded that the degradation rate of *DBB* segments is higher than *SEB* segments.

**Figure 5:**
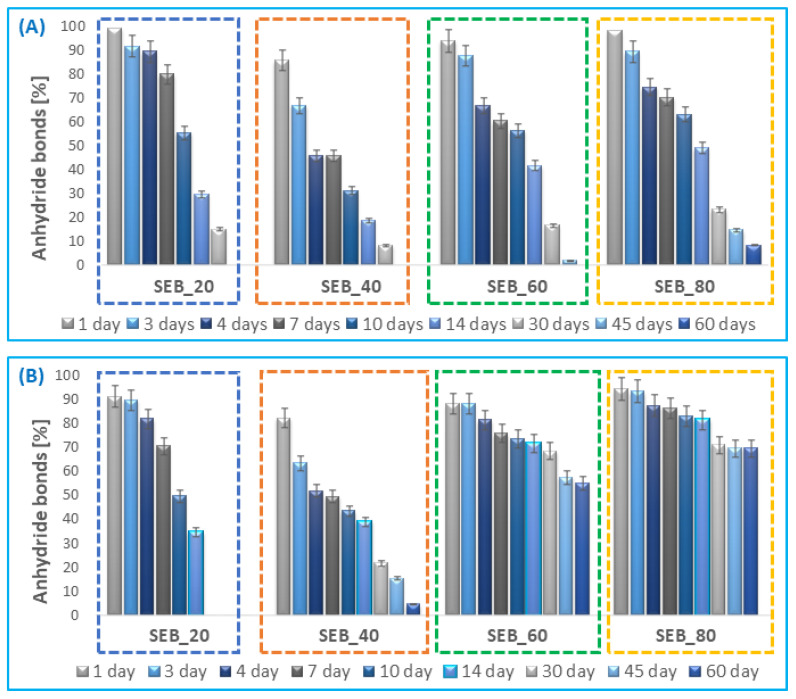
Anhydride bond loss of *DBB*-*SEB* polyanhydrides, for *DBB* segments (**A**) and SA segments (**B**) in the air at 25 °C (n = 3, error bars, standard deviation).

In this work, the rate of hydrolytic degradation in air at room temperature was also investigated in order to check the stability of the polyanhydrides. In this study, the loss of anhydride bonds was determined after a certain time of keeping the sample in air (air humidity during the test was in the range of 40–50%) (Figure 5).

The degradation rate of *DBB* segments in copolymers in the air also increased with the increase in *DBB* content (Figure 5A). Similarly to the degradation results in PBS, *DBB* segments degrade faster than *SEB* segments (Figure 5B). The copolymers obtained with a 20 and 40% SA degraded completely (both *DBB* and *SEB* segments) within about 60 days, whereas for DBB_SEB_60 and DBB_SEB_80 the disappearance of anhydride bonds after 45 days was about 90–97% for *DBB* segments and about 30–45% for *SEB* segments.

### 3.3. Blank Microspheres Preparation and Characterization

**Figure 6:**
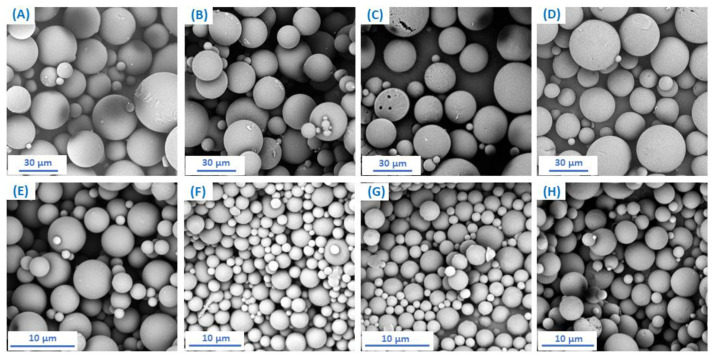
Scanning electron microscope (SEM) images of microspheres obtained from: DBB_SEB_20 (**A**,**E**); DBB_SEB_40 (**B**,**F**); DBB_SEB_60 (**C**,**G**) and DBB_SEB_80 (**D**,**H**), where (**A**–**D**) are large particles and (**E**–**H**) are small particles.

In order to test the usefulness of copolymers based on *DBB* and SA as carriers of biologically active compounds, attempts were made to obtain polymer microspheres. Microspheres were prepared by an emulsion (O/W) solvent evaporation technique while using poly(vinyl alcohol) as a stabilizing agent. By changing the speed of homogenization, it was possible to form spherical particles with smooth surfaces with diameters from 15–20 μm (for large particles obtained at 3000 rpm) and 2–5 μm (for small particles obtained at 18,000 rpm) (Figure 6, Table 5). The preparation of stable microspheres was possible for all obtained copolymers.

The size and size distribution of microspheres were calculated from an optical microscope (Table 5). The obtained results show that the size of large microspheres increases with the increase of SA content in polyanhydrides (except for DBB_SEB_80 microspheres), unlike for small microspheres, where the particle size decreases with an increase of SA content).

### 3.4. Rifampicin Loaded Microspheres Preparation and Characterization

**Figure 7:**
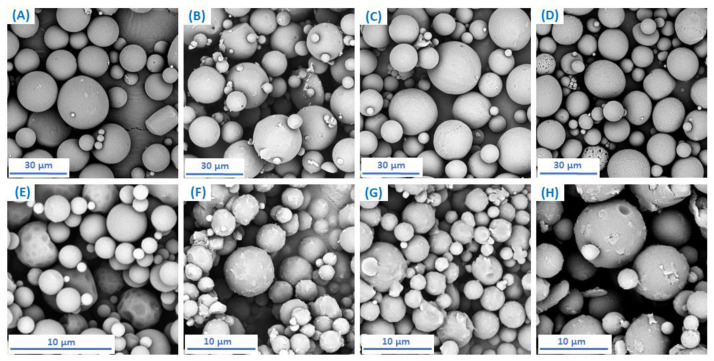
SEM images of RIF–MS obtained from: SEB_20_3 (**A**); SEB_40_3 (**B**); SEB_60_3 (**C**); SEB_80_3 (**D**); SEB_20_6 (**E**); SEB_40_6 (**F**); SEB_60_6 (**G**); SEB_80_6 (**H**).

In this study, 24 polymer-drug systems in which rifampicin was used as a biologically active compound were obtained. Rifampicin-loaded polyDBB_SEB microspheres (RIF–MS) were prepared by the emulsion-solvent evaporation method, similar to that used for the preparation of blank microspheres. Small (at 3000 rpm) and large (at 18,000 rpm) RIF–MS were obtained for each of the copolymers. The initial amount of drug was 10, 30 and 50 wt%, based on the weight of the polymer. The SEM images of RIF–MS (where initial amount of RIF was 50 wt%) are shown in Figure 7.

The characteristics of RIF–MS are collected in Table 6 (for large particles) and in Table 7 (for small particles). Obtained RIF–MS were round in shape with diameter in the range of 9–21 μm (large particles) and 1.8–5 μm (small particles) and of orange to red colour originating from rifampicin. The size and size distribution of microspheres were calculated from an optical microscope.

Parameters such as the actual rifampicin content in the microspheres, the encapsulation efficiency and drug loading were calculated using Equations (14)–(16). The encapsulation efficiency (*EE*) and drug loading (*DL*) were dependent on the polymer composition, particle size and the starting amount of the drug. Drug loading varied from 2% to 13%. It was found that drug loading increased with increasing the initial amount of RIF. The highest degree of *DL* was observed for large particles, in which the initial amount of drug (at the particle preparation stage) was 50 wt%. The composition of the polymer from which the polymer-drug systems were obtained also had a great influence on the degree of drug loading. The highest drug loading was observed for particles obtained from a copolymer containing 40–80 wt% of *DBB*. The least amount of drug that was introduced into the microspheres was obtained from a copolymer containing 80 wt% of sebacic acid. It can be concluded that the chemical structure of *DBB* influences the physical binding of rifampicin in polymer –drug systems. The RIF encapsulation efficacy varied from 7% to 33%. The *EE* for all copolymers was the highest when the initial amount of drug was 10 wt%, and the lowest when the initial amount of drug was 30 wt%.

**Figure 8:**
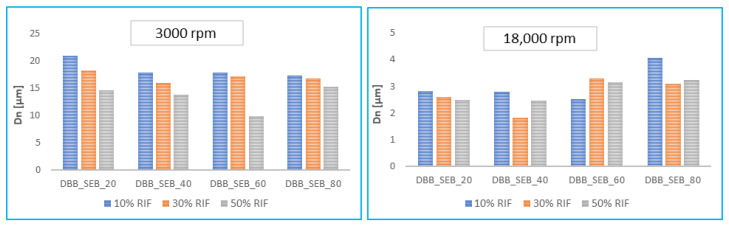
Comparison of the sizes of the RIF-loaded large particles (**left**) and small particles (**right**).

The size of microspheres was also dependent on the degree of loading of the microspheres with rifampicin (RIF-MS). RIF–MS size decreased with the increasing of RIF content in particles (Figure 8). It was most noticeable for the large particles. Microspheres with the highest diameters were obtained from DBB_SEB_20 for large particles and DBB_SEB_80 for the small particles. Large microspheres containing RIF were slightly smaller than respective blank ones. However, small RIF–MS were slightly smaller than the blank ones when the content of SA in copolymers was 20 and 40 wt%, but were larger for higher content of SA (DBB_SEB_60 and DBB_SEB_80) in RIF–MS. It can be concluded that the crystallinity of polyanhydrides influences the size of small drug-loaded particles.

Coagulation behaviour of microspheres has been examined by measuring zeta potential. Zeta potential (ZP) of small blank and RIF-loaded (initial amount of RIF %) microspheres are presented in Table 8.

ZP values measured for blank and rifampicin-loaded microspheres were within the ranges from −16.4 to −26.7 mV and from −10.5 to −20.8 mV, respectively. ZP values of blank particles were slightly higher compared to those of RIF-MS. The RIF loading did not significantly change the zeta potential and therefore it can be inferred that loading the microspheres with the drug had no effect on the surface area.

**Figure 9:**
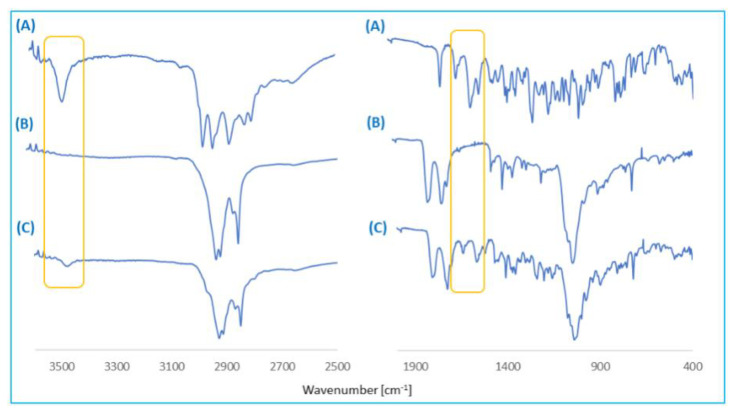
FT-IR spectra of rifampicin (**A**), copolymer (**B**) and rifampicin-loaded microspheres (**C**).

The FT-IR spectra of rifampicin, copolymer, blank and RIF-loaded microspheres were recorded and used to verify the loading of RIF in the poly(betulin disuccinate-*co*-sebacic acid) microspheres (Figure 9).

The RIF–MS spectra show characteristic absorption bands of RIF at 1643 cm^−1^, 1568 cm^−1^ and 1522 cm^−1^, which is absent in the FT-IR spectrum of unloaded microspheres. The appearance of characteristic peaks in the FT-IR spectrum of RIF–MS confirms the loading of rifampicin in microspheres. However, the peak sizes are smaller, which could be due to smaller amounts of rifampicin entrapped in the polymer matrix. The FT-IR results of RIF–MS discovered no new bands, indicating no chemical reaction between drug and polymer and suggesting a physical interaction between polyDBB_SEB and rifampicin.

**Figure 10:**
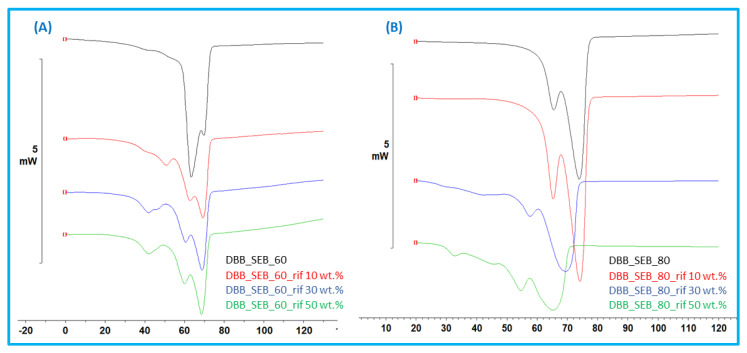
Representative DSC thermograms of RIF–MS obtained from: DBB_SEB_60 (**A**) and DBB_SEB_80 (**B**) with different content of RIF.

To check for any possible interaction between rifampicin and polyanhydrides, a compatibility study using DSC was carried out. DSC thermograms were performed for copolymer, RIF, blank particles and RIF–MS. The presence of rifampicin in microspheres caused a decrease in melting temperature (Tm) of crystalline polyanhydrides compared to Tm for blank microspheres (Figure 10).

It can be seen that the amount of drug incorporated in the microspheres influences the change in Tm. The higher the degree of rifampicin loading, the greater the change in Tm. The change in melting temperature was also more noticeable for DBB_SEB_80 than DBB_SEB_60. DSC results indicated that there was physical interaction between RIF and copolymer. Both FT-IR and DSC results confirm that there was no chemical reaction between drug and polymer.

### 3.5. In Vitro Drug Release

**Figure 11:**
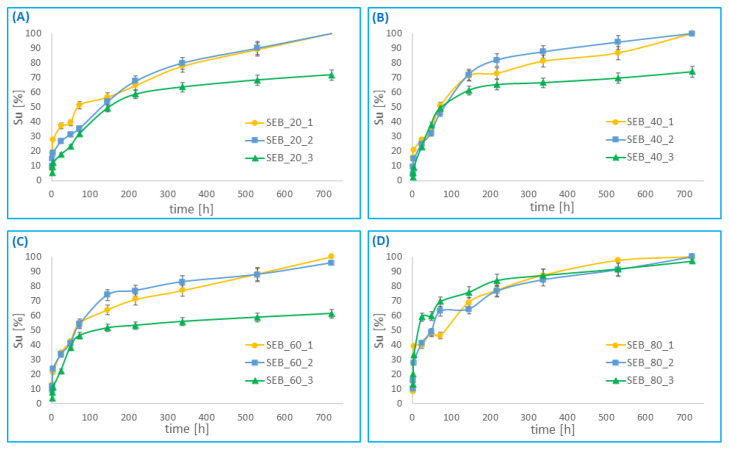
Cumulative release of rifampicin from large microspheres, obtained from: DBB_SEB_20 (**A**), DBB_SEB_40 (**B**), DBB_SEB_60 (**C**) and DBB_SEB_80 (**D**), as a function of time.

**Figure 12:**
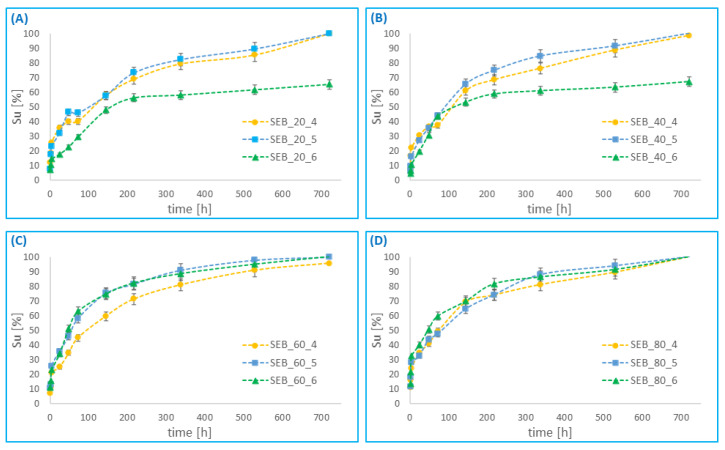
Cumulative release of rifampicin from small microspheres, obtained from: DBB_SEB_20 (**A**), DBB_SEB_40 (**B**), DBB_SEB_60 (**C**) and DBB_SEB_80 (**D**), as a function of time.

Drug release studies were carried out on the different RIF–MS prepared from poly(betulin disuccinate-*co*-sebacic acid). The RIF release profile for 30 days dissolution is presented in Figure 11 for large RIF–MS, and in Figure 12 for small RIF–MS. Rifampicin was released from the microspheres for a relatively long time (about 1 month for most systems). The rate of rifampicin release from the microspheres depends on the degree of drug loading (the more drug loaded, the longer rifampicin is released from the microspheres), and on the composition of the polyanhydride from which the microspheres were obtained. The rifampicin release from microspheres was rapid, within 72 h in most polymer—drug systems. In this time, about 40–60% of the total amount of encapsulated rifampicin was released. Slower, gradual release of RIF was observed within the next 25 days. For the large microspheres, the effect of the type of polyanhydride on the release profile is more apparent than for the small microspheres. The sebacic acid content of the polyanhydrides influenced the duration of the first release period (rapid release). For copolymers containing 80% of SA, the first period, in which 40–60% of RIF is released, is significantly shorter than for the other copolymers. This effect was less visible for the smaller particles.

The rate of release of rifampicin was slower than the rate of hydrolytic degradation of polyanhydrides for all polymer-drug compositions. This may be due to the deposition of poorly soluble degradation products (such as SA and *DBB*) on the surface of the microspheres, limiting the release of rifampicin. Additionally, the content of rifampicin in the particles also influenced the release rate. RIF release was slower in the case of RIF–MS, where the initial amount of RIF was 50%. After 30 days, only about 55–70% of rifampicin was released, while other RIF–MS released rifampicin completely within 30 days. It can be concluded that the higher the rifampicin content, the longer the drug was released for most systems. The case of rifampicin release from particles obtained from DBB_SEB_80 and DBB_SEB_60 was different. For these polymer-drug systems, the amount of rifampicin does not significantly affect the release rate. The obtained results indicate that the release of rifampicin from microspheres prepared from copolymer containing high content of sebacic acid is relatively faster and more corresponding to the hydrolytic degradation of polymers.

### 3.6. Kinetics of Rifampicin Release

To understand the mechanism of rifampicin release from polyDBB_SEB microspheres, the drug release data was plotted into the Korsmeyer-Peppas equation (Equation (20)) as log cumulative percentage of drug released versus log time, and the value of the diffusion exponent (n) was calculated using the slope of the straight line. When the release mechanism is not well known, such a model helps to identify which types of release phenomena are involved [31]. Values of exponent n = 0.5 or less correspond to a Fickian diffusion mechanism, 0.5 < n <1.0 to non-Fickian transport or anomalous diffusion, n = 1.0 to case II (relaxational) transport or typical zero order release, and n > 0.89 to super case II transport. For spherical drug carriers, the threshold of the n values distinguishing between Fickian and non-Fickian diffusion mechanism has been slightly modified and thus n values between 0.43 and 0.85 represent anomalous transport [33,34]. Kinetic parameters K and n for rifampicin–loaded microspheres are summarized in Table 9.

Equation (20) is valid for the first 60% of the release, especially when diffusion plays an important role in the release mechanism. The value of the diffusion exponent n, determined for almost all of rifampicin-loaded microspheres, ranged from 0.27 to 0.48, indicating that the release mechanism of rifampicin is Fickian–diffusion controlled. An exception is the large particles obtained from DBB_SEB_40, for which the value of the diffusion exponent n was equal to 0.64, indicating a non-Fickian diffusion mechanism.

The in vitro release data was also subjected to various release models, including zero order, first order, Higuchi and Korsmeyer-Peppas models. Regression analysis was performed to obtain the R^2^ (coefficient of correlation) values of the linear curves and the rate constants. In order to provide better understanding of kinetics, the release data was split as 0–4 h and 24–720 h. The kinetics of the first stage of release (0–4 h) for particles with an initial amount of drug 10 wt% depended on the particle size and composition of the copolymers. For amorphous copolymers (DBB_SEB_20 and DBB_SEB_40), drug release from large particles followed mainly first order kinetics, and the drug release from small particles was according to the Higuchi model. In the case of crystalline polyanhydrides (DBB_SEB_60 and DBB_SEB_80), drug release from large particles followed the Higuchi model for DBB_SEB_60, and zero order for DBB_SEB_80. However, drug release from small particles followed first order for DBB_SEB_60 and Korsmeyer-Peppas for DBB_SEB_80. The kinetics of the first release stage (0–4 h) for particles with initial amount of drug 30 wt%, drug release followed first order kinetics, and for particles with initial amount of drug 50 wt%, the Korsmeyer-Peppas kinetics model. A different result was obtained for DBB_SEB_20 where, regardless of the initial amount of the drug and particle size, the drug release followed the Higuchi model.

The results obtained for the second stage of release (24–720 h) indicate that the release kinetics does not depend on the particle size and the composition of polymer. For microspheres with an initial amount of drug 10 and 50 wt%, the kinetics mainly followed the Korsmeyer-Peppas model; however, for particles with an initial amount of drug 30 wt%—first order kinetics.

### 3.7. Antibacterial Activity

In order to evaluate the efficacy of the microspheres on biological systems, the antibacterial activity of selected blank and rifampicin-loaded microspheres against *Staphylococcus aureus* (ATCC 25923) bacteria was examined. For this study, blank and RIF-loaded microspheres (loaded with the greatest amount of RIF) obtained from two copolymers (containing 20 and 80 wt% of sebacic acid) were chosen.

**Figure 13:**
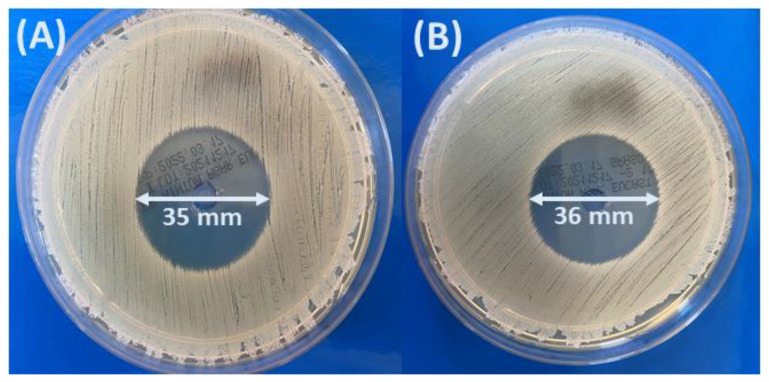
Inhibition zones of *Staphylococcus aureus* (ATCC 25923) after culture with the rifampicin released from the SEB_20_3 (**A**) and SEB_80_3 (**B**) microspheres after 24 h into the PBS.

In the case of RIF-loaded microspheres, inhibition of bacterial growth was observed, whereas for blank particles, no inhibition effect was observed. The concentration of the drug released from microspheres after 24 h was enough to inhibit bacteria. Figure 13 presents *Staphylococcus aureus* growth inhibition zones.

For rifampicin-loaded microspheres, the inhibition zone was 35 mm for SEB_20_3 and 36 mm for SEB_80_3 microspheres. Our results indicate high antimicrobial activity; inhibited zone is much higher compared with the results presented in [35], where it was reported that inhibition zone of S. aureus only for rifampicin was near 9 mm. However, for propolis particles with rifampicin, the inhibition zones were higher, between 9 and 17 mm. 

## 4. Conclusions

Biodegradable polymers such as polyanhydrides are useful in drug delivery applications because of their lack of toxicity and the appropriate release kinetics of active substances. In the course of this study, new biodegradable polyanhydrides composed of betulin disuccinate and sebacic acid were obtained and used to formulate small and large microspheres using the emulsion solvent evaporation method. Rifampicin, an ansamycin drug, was encapsulated into the microspheres. Obtained RIF–MS were round in shape with diameter in the range of 9–21 μm for large particles and 1.8–5 μm for small particles and of orange to red colour originating from rifampicin. The encapsulation efficiency and drug loading were dependent on the polymer composition, particle size and the starting amount of the drug. The RIF encapsulation efficacy varied from 7% to 33%. Drug loading varied from 2% to 13% and increased at a higher RIF ratio. Rifampicin was released from polyDBB_SEB microspheres for a relatively long time (about 1 month for most systems); however, about 40–60% of the drug was released within the first 72 h. The rate of release of rifampicin was slower than the rate of hydrolytic degradation of polymers for all polymer-drug composition, but the releasing of rifampicin from microspheres prepared from copolymer containing a high content of sebacic acid was relatively faster and more corresponding to hydrolytic degradation of polymers. The drug release mechanism for almost all polymer-drug systems corresponded with Fickian diffusion. The kinetics of rifampicin release depended on the particle size and composition of the copolymers only when the initial amount of drug was 10 wt%. The antibacterial effect of RIF-loaded microspheres was confirmed by tests with *Staphylococcus aureus* (ATCC 25923) bacteria. The concentration of loaded drug was enough to obtain bactericidal effects. Obtained results indicate that the poly(betulin disuccinate-*co*-sebacic acid) microspheres prepared in this study serve as promising drug delivery systems for rifampicin.

## Figures and Tables

**Table 1:** Feed ratio of *DBB* and *SEB*.

Polyanhydride	Feed Ratio [% *w*/*w*]		Feed Ratio *DBB*:*SEB* [mol/mol]
	*DBB*	*SEB*	
polyDBB	100	0	—
DBB_SEB_20	80	20	1:0.79
DBB_SEB_40	60	40	1:2.12
DBB_SEB_60	40	60	1:4.77
DBB_SEB_80	20	80	1:12.71
PSA	0	100	—

**Table 2:** Rifampicin loaded microspheres.

Large Microspheres 3000 rpm			Small Microspheres 18,000 rpm		
Sample	SA Content [% *w*/*w*]	Rif. Cont. [% *w*/*w*]	Sample	SA Content [% *w*/*w*]	Rif. Cont. [% *w*/*w*]
SEB_20_1	20	10	SEB_20_4	20	10
SEB_20_2		30	SEB_20_5		30
SEB_20_3		50	SEB_20_6		50
SEB_40_1	40	10	SEB_40_4	40	10
SEB_40_2		30	SEB_40_5		30
SEB_40_3		50	SEB_40_6		50
SEB_60_1	60	10	SEB_60_4	60	10
SEB_60_2		30	SEB_60_5		30
SEB_60_3		50	SEB_60_6		50
SEB_80_1	80	10	SEB_80_4	80	10
SEB_80_2		30	SEB_80_5		30
SEB_80_3		50	SEB_80_6		50

**Table 3:** Characteristics of *DBB*-*SEB* polyanhydrides.

Polyanhydride	Feed Ratio *DBB*:SA [mol/mol]	*DBB*:SA in Polymer [mol/mol] Calculated from ^1^H NMR	Mn (^1^H NMR)	Molecular Weight (GPC)			DSC		
				M_n_	M_w_	DP	Tg [°C]	Tm [°C]	ΔHm [J/g]
polyDBB	—	—	8200	8500	25,000	2.94	124.0	—	—
DBB_SEB_20	1:0.79	1:0.78	11,000	7100	23,100	3.24	85.6	—	—
DBB_SEB_40	1:2.12	1:2.12	11,000	7600	24,900	3.29	22.5	—	—
DBB_SEB_60	1:4.77	1:4.76	13,400	11,500	50,900	4.41	—	41.1; 68.0	−9.03; −27.27
DBB_SEB_80	1:12.71	1:12.41	15,000	13,000	45,200	2.24	36.8	80.3	−74.96
PSA	—	—	10,000	10,800	21,600	2.06	—	80.8	−98.04

*DBB*:*SEB* in polymer (^1^H NMR) and Mn (^1^H NMR) were calculated according to Equations (1)–(6): Tg—glass temperature of polymers determined as a midpoint of glass transition, Tm—melting temperature and ΔHm—heat of fusion of melting process.

**Table 4:** Solubilities of DBB-*SEB* polyanhydrides.

Polyanhydride	Acetone	H_2_O	EtOH	Toluene	Diethyl Ether	THF	DMSO	CHCl_3_	CH_2_Cl_2_	Hexane
polyDBB	—	—	—	+	—	+	±	+	+	—
DBB_SEB_20	±	—	—	+	—	+	±	+	+	—
DBB_SEB_40	±	—	—	+	—	+	±	+	+	—
DBB_SEB_60	±	—	—	+	—	+	+	+	+	—
DBB_SEB_80	±	—	—	+	—	+	+	+	+	—
PSA	±	—	—	+	—	—	±	+	+	—

+ soluble, ± partially soluble, — insoluble.

**Table 5:** Size and size distribution of blank microspheres calculated from optical microscope.

Polyanhydride	Homogenizer rpm	*D_n_* [μm]	SD	*D_v_*/*D_n_*
DBB_SEB_20	3000	15.70	6.31	1.44
	18,000	5.17	2.94	1.91
DBB_SEB_40	3000	18.20	6.18	1.44
	18,000	3.79	2.53	2.78
DBB_SEB_60	3000	20.17	9.38	1.53
	18,000	2.88	0.89	1.29
DBB_SEB_80	3000	17.90	6.34	1.29
	18,000	1.98	0.49	1.16

*D_n_*—number average diameters, *D_v_*—volume average diameters, SD—standard deviation and *D_v_*/*D_n_*—dispersity index.

**Table 6:** Microsphere size, actual and theoretical loading, encapsulation efficiency and drug loading of large rifampicin-loaded microspheres.

Sample	*D_n_* ± SD	*D_v_*/*D_n_*	*L_A_* ± SD [μg/mg]	*L_Th_* [μg/mg]	*EE* ± SD [%]	*DL* ± SD [%]
SEB_20_1	20.88 ± 9.61	1.39	23.8 ± 0.9	100	23.8 ± 0.7	2.38 ± 0.3
SEB_20_2	18.18 ± 7.17	1.44	45.6 ± 1.5	300	15.2 ± 0.4	4.56 ± 0.7
SEB_20_3	14.64 ± 7.30	1.62	104.6 ± 3.1	500	20.9 ± 0.5	10.46 ± 1.2
SEB_40_1	17.89 ± 10.02	1.67	32.9 ± 1.1	100	32.9 ± 1.4	3.29 ± 0.5
SEB_40_2	16.01 ± 8.18	1.55	39.8 ± 0.9	300	13.3 ± 0.5	3.98 ± 0.5
SEB_40_3	13.74 ± 7.51	1.73	103.3 ± 2.9	500	20.7 ± 1.0	10.33 ± 0.9
SEB_60_1	17.90 ± 8.30	1.48	28.9 ± 0.7	100	28.9 ± 1.1	2.89 ± 0.4
SEB_60_2	17.15 ± 9.28	1.61	38.5 ± 1.2	300	12.8 ± 0.3	3.85 ± 0.6
SEB_60_3	9.92 ± 4.82	1.58	122.3 ± 2.6	500	24.5 ± 0.9	12.23 ± 1.4
SEB_80_1	17.27 ± 6.19	1.32	22.1 ± 0.4	100	22.1 ± 0.8	2.71 ± 0.4
SEB_80_2	16.72 ± 8.61	1.54	33.7 ± 1.3	300	11.2 ± 0.4	3.37 ± 0.9
SEB_80_3	15.32 ± 8.38	1.70	61.7 ± 1.7	500	12.3 ± 0.5	6.17 ± 1.0

*D_n_*—number average diameters, *D_v_*—volume average diameters, SD—standard deviation, *D_v_*/*D_n_*—dispersity index, *EE*—encapsulation efficiency, *L_A_*— actual RIF loading, *L_Th_*—theoretical RIF loading and *DL*—drug loading. Microspheres sizes were calculated from optical microscope.

**Table 7:** Microsphere size, actual and theoretical loading, encapsulation efficiency and drug loading of small rifampicin-loaded microspheres.

Sample	*D_n_* ± SD	*D_v_*/*D_n_*	*L_A_* ± SD [μg/mg]	*L_Th_* [μg/mg]	*EE* ± SD [%]	*DL* ± SD [%]
SEB_20_4	2.81 ± 1.30	1.57	27.5 ± 1.1	100	27.5 ± 0.8	2.75 ± 0.2
SEB_20_5	2.60 ± 1.19	1.59	32.7 ± 1.8	300	10.9 ± 0.5	3.27 ± 0.7
SEB_20_6	2.48 ± 1.09	1.48	90.2 ± 3.2	500	18.0 ± 0.7	9.02 ± 1.1
SEB_40_4	2.78 ± 1.15	1.43	31.6 ± 1.5	100	31.6 ± 1.3	3.16 ± 0.9
SEB_40_5	1.81 ± 0.71	1.36	34.7 ± 1.3	300	11.6 ± 0.7	3.47 ± 1.2
SEB_40_6	2.46 ± 0.99	1.54	87.7 ± 3.4	500	17.5 ± 0.9	8.77 ± 1.3
SEB_60_4	2.51 ± 0.83	1.28	22.8 ± 0.8	100	22.8 ± 1.1	2.28 ± 0.3
SEB_60_5	3.28 ± 1.25	1.43	31.4 ± 1.0	300	10.5 ± 0.3	3.14 ± 0.5
SEB_60_6	3.14 ± 1.33	1.57	54.1 ± 3.1	500	10.8 ± 0.5	5.41 ± 0.9
SEB_80_4	4.05 ± 1.50	1.31	22.6 ± 1.4	100	22.6 ± 0,8	2.26 ± 0.5
SEB_80_5	3.10 ± 1.35	1.53	21.3 ± 1.1	300	7.1 ± 0.2	2.13 ± 0.2
SEB_80_6	3.24 ± 1.46	1.52	39.6 ± 2.1	500	7.9 ± 0.1	3.96 ± 1.0

*D_n_*—number average diameters, *D_v_*—volume average diameters, SD—standard deviation, *D_v_*/*D_n_*—dispersity index, *EE*—encapsulation efficiency, *L_A_*— actual RIF loading, *L_Th_*—theoretical RIF loading and *DL*—drug loading. Microsphere sizes were calculated from optical microscope.

**Table 8:** Comparison of the zeta potential of small blank and RIF loaded (50%) microspheres.

Blank Microspheres		RIF Loaded Microspheres	
Sample	ZP ± SD [mV]	Sample	ZP ± SD [mV]
SEB_20	−26.7 ± 0.49	SEB_20_6	−20.8 ± 0.1
SEB_40	−16.4 ± 1.88	SEB_40_6	−10.5 ± 2.1
SEB_60	−24.8 ± 0.8	SEB_60_6	−20.1 ± 1.3
SEB_80	−20.2 ± 0.94	SEB_80_6	−18.7 ± 1.14

ZP—zeta potential, SD—standard deviation.

**Table 9:** Kinetic parameters K and n for RIF–MS, calculated according to Equation (20).

Initial Amount of Drug								
10 wt%			30 wt%			50 wt%		
RIF–MS	K	n	RIF–MS	K	n	RIF–MS	K	n
SEB_20_1	12.05	0.34	SEB_20_2	10.93	0.29	SEB_20_3	5.89	0.40
SEB_20_4	14.55	0.27	SEB_20_5	10.98	0.35	SEB_20_6	7.47	0.35
SEB_40_1	7.46	0.44	SEB_40_2	5.60	0.48	SEB_40_3	3.00	0.64
SEB_40_4	10.82	0.33	SEB_40_5	6.84	0.44	SEB_40_6	4.90	0.47
SEB_60_1	11.58	0.35	SEB_60_2	10.11	0.38	SEB_60_3	5.47	0.43
SEB_60_4	8.49	0.38	SEB_60_5	11.31	0.38	SEB_60_6	11.76	0.38
SEB_80_1	12.48	0.35	SEB_80_2	12.27	0.38	SEB_80_3	15.50	0.39
SEB_80_4	12.90	0.31	SEB_80_5	13.94	0.30	SEB_80_6	16.48	0.30

## Data Availability

The data presented in this study are available on request from the corresponding author.
